# IFI204-STING drives protective innate immunity against gangrenous *Clostridium perfringens* infection via regulation of NLRP3 signaling

**DOI:** 10.3389/fimmu.2026.1715595

**Published:** 2026-03-31

**Authors:** Ming-Yue Zhang, Jia-Qi Li, Qian Xu, Zi-Jian Zhuang, Jing Liang, Ao-Bo He, Shu-Xin Zhang, Yi Zhao, Xue Chen, Zhen-Yu Li, Ping Sheng, Yang Liu, Shui-Xing Yu

**Affiliations:** 1 State Key Laboratory of Reproductive Regulation and Breeding of Grassland Livestock, College of Life Sciences, Inner Mongolia University, Hohhot, China; 2 Institute of Animal Husbandry, Inner Mongolia Academy of Agricultural and Animal Husbandry Sciences, Hohhot, China

**Keywords:** *Clostridium perfringens*, gas gangrene, Ifi204, innate immune, NLRP3 inflammasome, STING

## Abstract

Inflammasomes are an essential component of the innate immune response against pathogen infections. However, the molecular mechanism regulating the inflammasome signaling in response to gangrenous *Clostridium perfringens* (*C. perfringens*) infection remains elusive. We herein report the unexpected discovery that IFI204 (the murine homolog of human IFI16)–dependent STING protects against *C. perfringens* gas gangrene via enhancing NLRP3 inflammasome signaling. In the *C. perfringens* gas gangrene model, compared with wild-type (WT) mice, *Sting* deficiency (*Sting^-/-^*) mice displayed an increased susceptibility to *C. perfringens* soft tissue infection, with more bacterial colonization, severe muscle damage, and higher mortality rates. Obviously, *Sting* deficiency leads to the defect of inflammasome signaling activation and bacterial killing and clearance. STING promotes inflammasome signaling activation in an IFI204-dependent manner. Crucially, the IFI204-STING axis enhances NLRP3 inflammasome signaling activation, which, in turn, facilitates pathogen elimination and host defense. Our findings highlight STING acts as a positive regulator in defense of *C. perfringens* infection and present it as a potential target for anti-infection drug development.

## Introduction

*C. perfringens* can cause explosive fulminant necrotizing infections and is a common cause of gas gangrene or muscle necrosis (1, 2), posing a significant problem to both animal husbandry and human health (3). The key feature of these diseases is that they are mediated by the production of potent toxins and extracellular enzymes (4, 5). At least 20 toxins have been reported as produced by *C. perfringens*, of which α and θ toxins are essential for gas gangrene (6–8). Hitherto, traumatic injury contributes to 70% of gas gangrene cases, of which 80% are caused by *C. perfringens* (9, 10). However, the treatment recommendation for severe *C. perfringens* gas gangrene is still radical amputation or the use of high doses of antibiotics, a combination of penicillin with clindamycin or carbapenem (11–13). Presently, with the emergence of drug-resistant strains and growing scientific concern over the resistance spectrum, the novel preventive and therapeutic strategies for the control of *C. perfringens* infections have become more urgent.

Host innate immunity is critical for detecting invading pathogens, triggering anti-infection defenses, and facilitating pathogen clearance (14–16). Sensing and categorizing pathogen invaders via a majority of germline-encoded pattern recognition receptors, including NOD-like receptors, cytosolic DNA sensors, toll-like receptors, C-type lectin receptors, and RIG-I like receptors, which in turn initiates appropriate immune responses that mobilize defense mechanisms (17–20). Concerning *C. perfringens*, TLR2, TLR4, AIM2, and NLRP3, were involved in *C. perfringens* recognition (21–24). Also, we provided evidence indicating that the free fatty acid receptor GPR120 and the necroptosis effector MLKL contribute to host protection against *C. perfringens* infection by enhancing NLRP3 inflammasome signaling and extracellular trap formation (12, 25). Therefore, deeper research on innate immune mechanisms of host defense is expected to open new avenues for therapeutic interventions of pathogenic infectious diseases.

STING, also named TMEM173, STING1, ERIS, MITA, or MPYS, is a pivotal cyclic dinucleotide sensor (26–28) and adaptor protein for various DNA sensors such as IFI204 (IFI204, the murine homolog of human IFI16), cGAS, DDX41, and ZBP1 (29–31). In past decades, emerging evidence shows STING is associated with health and disease by regulating innate immune responses, inflammatory signaling, cell death, and host-pathogen interaction (32–35). As an endoplasmic reticulum-associated transmembrane protein, STING can be directly initiated by microbial cyclic dinucleotides (including cyclic-di-GMP and cyclic-di-AMP) or the second messenger cGAMP, a process that participates in inflammation signaling and immune response by producing IFNs and pro-inflammatory mediators. Indeed, it was previously reported that the effect of STING on pathogenic infectious diseases is controversial, which is not only beneficial to host protection against *S. aureus*, *M. tuberculosis*, *S. pneumoniae*, *P. aeruginosa*, *Brucella*, and *L. monocytogenes* (36–41) but also detrimental to the host during *S. aureus* cutaneous infection may depend on diverse pathogens and different infectious models (42). However, there is little knowledge regarding the biological implications of STING in response to *C. perfringens* infection.

In this study, we deliberated whether STING is involved in host defense against *C. perfringens* gas gangrene. The collected data here demonstrate that the active STING promotes NLRP3 inflammasome signaling-mediated bacterial killing and clearance. Activated STING downstream of IFI204 confers host resistance to gangrenous *C. perfringens* infection. These findings reveal a scaffolding role of STING in enhancing protective innate immunity against *C. perfringens*, which provides further insight into host-pathogen interaction mechanisms and innovative strategies targeting pathogenic infectious diseases.

## Materials and methods

### Experimental animals and cells

*Ifi204* deficiency (*Ifi204^-/-^*) and *Sting^-/-^* mice were gifts from Dr. Yong-Jun Yang (Jilin University, China). *Nlrp3* deficiency (*Nlrp3^-/-^*) and C57BL/6J (WT) mice were obtained from Jackson Laboratory (Bar Harbor, ME, USA). The mice were maintained in a pathogen-free environment at the Laboratory Animal Center of Inner Mongolia University, and all experiments adhered to Animal Research: Reporting of *In Vivo* Experiments (ARRIVE) guidelines (43) and were conducted according to experimental practices and standards approved by the Animal Welfare and Research Ethics Committee at Inner Mongolia University ([2022] 071). Murine macrophages (bone marrow–derived macrophages, BMDMs) were isolated from the femurs of 6- to 8-week-old mice, cultured, and matured in a certain medium, RPMI1640 (Gibco, #31800-022), fetal bovine serum (Gibco, #A31608-02), and L929 cell–conditioned supernatant supplemented with penicillin/streptomycin (Gibco, #15140-122) as previously described (12).

### Gas gangrene model

The *C. perfringens* gas gangrene model was established as previously reported (25), and the experiments complied with the manual of the care and use of laboratory animals published by the US National Institutes of Health. In brief, age- and sex-matched mice were maintained under general anesthesia with isoflurane and were intramuscularly challenged with 1 × 10^7^ CFUs of log-phase pathogenic *C. perfringens* strain CP1 (a clinical isolate, NCBI GenBank: MW440585). At 24 hp.i., infected mice were euthanized by IV injection with sodium pentobarbital, and homogenized muscle, liver, and spleen tissues were plated onto BHI agar plates (Haibo Biotechnology, #HB8297-5) for bacterial loads (CFUs/g) to be enumerated. For the survival study, age- and sex-matched mice were intramuscularly infected with 5 × 10^7^ CFUs of log-phase pathogenic *C. perfringens* strain CP1, and the mortality of mice were monitored. The pathology scoring criteria of gas gangrene were determined according to the severity of blackening, limping, grip, stiffness, malaise, deformation, and swelling (each item scores from 0 to 3) as previously described (43).

### Inflammasome activation

Matured BMDMs were pre-primed with LPS (500 ng/ml, 4h, Invitrogen, #tlrl-3pelps) and then incubated/infected with ATP (5 mM, 30 min, Sigma, #A2383), *C. perfringens* strain CP1 (NCBI GenBank: MW440585) or strain ATCC13124 (MOI = 20, 90 min). Cell samples were obtained for ELISA, immunostaining, and immunoblotting detections.

### Bacterial killing assessment

LPS-primed mature BMDMs were pre-treated with recombinant IL-18 (rIL-18, 1 ng/ml, 1h, Novoprotein, #CK06) or equal PBS prior to challenge with *C. perfringens* strain CP1 (NCBI GenBank: MW440585) or strain ATCC13124 (MOI = 5, 6h). The cell supernatants were harvested and plated on BHI agar plates, and bacterial loads (CFUs/ml) were counted.

### RNA sequencing

LPS-primed matured BMDMs were infected with *C. perfringens* strain CP1 (NCBI GenBank: MW440585) or strain ATCC13124 (MOI = 20) for 90 min. The total cell RNA was then extracted, examined, generated double-stranded cDNA, and submitted for sequencing on an Illumina NovaSeq 6000 at Shanghai Personal Biotechnology Co., Ltd., China. The differentially expressed genes were estimated by calculating the fold change (log_2_ fold change > 1) and *p*-value (< 0.05).

### Histology and immunofluorescence

The infected leg muscle was aseptically harvested and fixed in 4% buffered formalin solution (Macklin, #P804536), and sections were stained with H&E (Solarbio, #SL7050-500) or immunofluorescence staining. Immunostaining, including muscle sections or stimulated BMDMs, was performed using anti-STING (Proteintech, #19851-1-AP), anti-F4/80 (BioLegend, #123119), anti-Gr-1 (Abcam, #ab196436), anti-IFI204 (Abcam, #ab307201), anti-ASC (Adipogen, #A29151803f), Alexa Fluor488–conjugated anti-rabbit IgG (Invitrogen, #ab150077), Alexa Fluor594–conjugated anti-mouse IgG (Invitrogen, #ab150116) antibodies, and DAPI (Solarbio, #C0065).

### Proinflammatory cytokines measurements

Cytokines and chemokines in the supernatants of homogenized muscle tissues or stimulated BMDMs were detected by ELISA according to the manufacturer’s instructions. The mouse IL-1β (Cat#MLB00C), mouse TNF-α (Cat#MTA00B), mouse IL-6 (Cat#PM6000B), and mouse KC (Cat#PMKC00B) ELISA kits were purchased from R&D Systems.

### Immunoblotting

The total lysates or supernatants of stimulated BMDMs or homogenized muscle tissues were separated by SDS-PAGE and transferred to a PVDF membrane. Immunoblotting was performed using anti-STING (Proteintech, #19851-1-AP), anti-Caspase-1 (Adipogen, #A28881708), anti-NLRP3 (AdipoGen, #AG-20B-0014), anti-IFI204 (Abcam, #ab307201), and anti-GAPDH (Proteintech, #60004-1-Ig).

### Statistical analysis

Quantification and statistical analysis were performed using GraphPad Software (La Jolla, CA), with a significance threshold set at *p* < 0.05 (*p < 0.05 or **p < 0.01). Error bars represent the mean ± standard deviation (SD). An unpaired Student’s t test was utilized for comparisons between two groups, while analysis of variance followed by Dunnett’s, Tukey’s, or log-rank test was employed for multiple group comparisons.

## Results

### Sting deficiency leads to a defect in bacterial clearance following gangrenous *C. perfringens* infection

**Figure 1:**
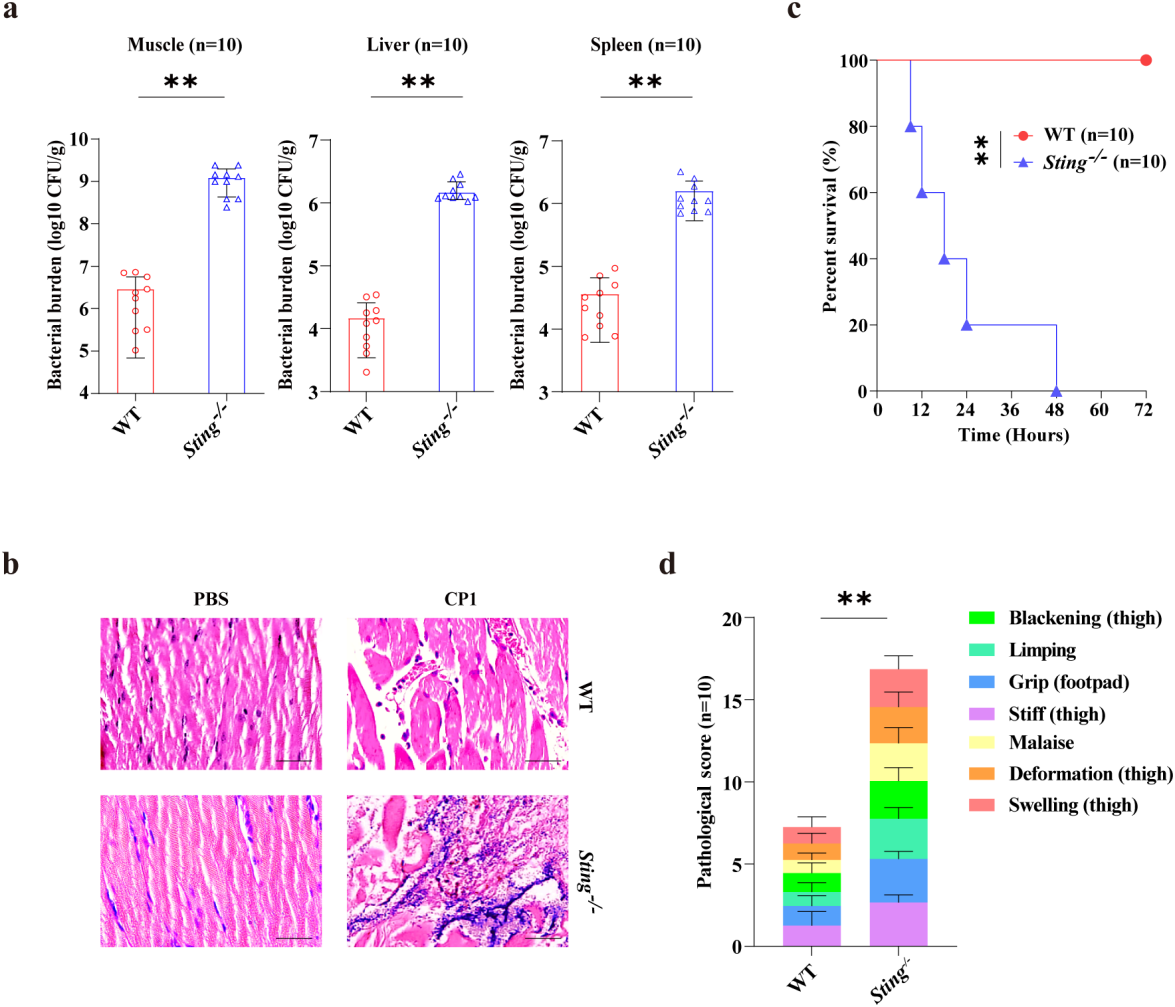
STING contributes to protecting against pathogenic *C. perfringens* soft tissue infection. Age- and sex-matched WT and *Sting^-/-^* mice (*n* = 10 each group) were intramuscularly challenged with log-phase *C. perfringens*. **(a)** Bacterial loads in the muscle, liver, and spleen were enumerated (1 × 10^7^ CFUs, at 24h p.i.). **(b)** Representative HE staining of the leg muscle tissue (1 × 10^7^ CFUs, at 24h p.i., magnification, ×400). **(c, d)** Survival and cumulative gross pathology scores of infected mice (5 × 10^7^ CFUs). Graphs are means ± standard deviation (SD) from data pooled ten **(a, c, d)** biological replicates. Data were considered significant when **p < 0.01.

To investigate the possible involvement of STING in the host response to *C. perfringens* soft tissue infection, a *C. perfringens* gas gangrene model was initially established. Littermate WT control mice and *Sting^-/-^* mice were intramuscularly infected with 1 × 10^7^ CFUs of gangrenous *C. perfringens* strain CP1 (NCBI GenBank: MW440585). At 24h p.i., bacterial loads were assessed. Notably, significantly increased bacterial counts were detected in the muscle, liver, and spleen of infected *Sting^-/-^* mice compared to infected WT counterparts (**Figure 1a**). In accordance with this, infected *Sting^-/-^* mice also exhibited severely impaired muscle architecture, suggesting that *Sting^-/-^* mice are more susceptible to gangrenous *C. perfringens* infection (**Figure 1b**). To gain additional evidence of the protective role of STING against gangrenous *C. perfringens* infection, the survival rate of WT and *Sting^-/-^* mice was monitored following the intramuscular administration of 5 × 10^7^ CFUs of gangrenous *C. perfringens*. The results showed that all of the *Sting^-/-^* mice had died at 48h p.i. along with obvious clinical symptoms, including limping, swelling, blackening, grip loss, deformation, intense stiffness, and malaise (**Figures 1c, d**). Thus, these data demonstrate that STING contributes to host protection against gangrenous *C. perfringens* infection.

### Sting deficiency attenuates inflammasome signaling activation during pathogenic *C. perfringens* soft tissue infection

**Figure 2:**
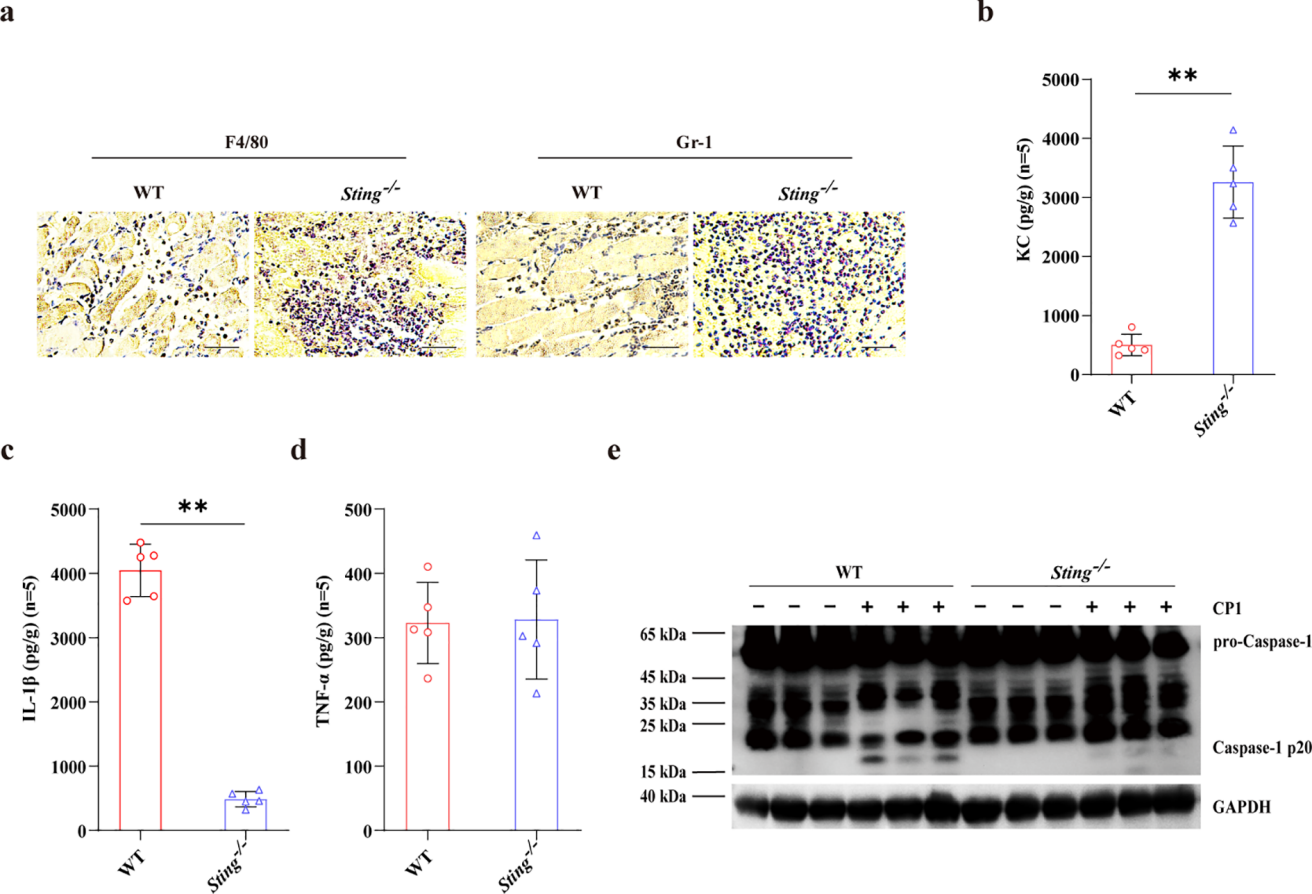
STING activates inflammasome signaling following gangrenous *C. perfringens* infection. Age- and sex-matched WT and *Sting*^-/-^ mice were intramuscularly stimulated with 1×10^7^ CFUs log-phase *C. perfringens* at 24 h p.i. **(a)** Representative infected muscle sections. F4/80 (IHC, a macrophages marker) and Gr-1 (IHC, a neutrophil marker) stained brown (magnification, ×400). **(b–d)** Secreted KC, IL-1β, and TNF-α in the homogenate supernatant of infected muscle were determined by ELISA. **(e)** Cleaved Caspase-1 in the muscle tissue lysate was examined by immunoblotting. Graphs are means ± standard deviation (SD) from data pooled five **(b–d)** biological replicates. Data were considered significant when **p < 0.01.

To further illustrate the potential immunological role of STING in defense of gangrenous *C. perfringens*, inflammatory response was initially determined by protein levels *in vivo*. Obviously, despite increased infiltration, including macrophages and neutrophils (PMNs), and elevated KC expression (**Figures 2a, b**), the IL-1β production was significantly attenuated in the muscles of *Sting^-/-^* mice compared to WT controls following pathogenic *C. perfringens* soft tissue infection (**Figure 2c**), whereas TNF-α release was comparable between both genotypes (**Figure 2d**). Moreover, inflammasome-dependent Caspase-1 activation was also significantly inhibited in the muscles of infected *Sting^-/-^* mice (**Figure 2e**; **Supplementary Figure 1**). These results revealed that *Sting* deficiency results in aberrant inflammation and impaired inflammasome signaling in coping with gangrenous *C. perfringens* infection.

### STING activates NLRP3 inflammasome signaling in response to pathogenic *C. perfringens*

**Figure 3:**
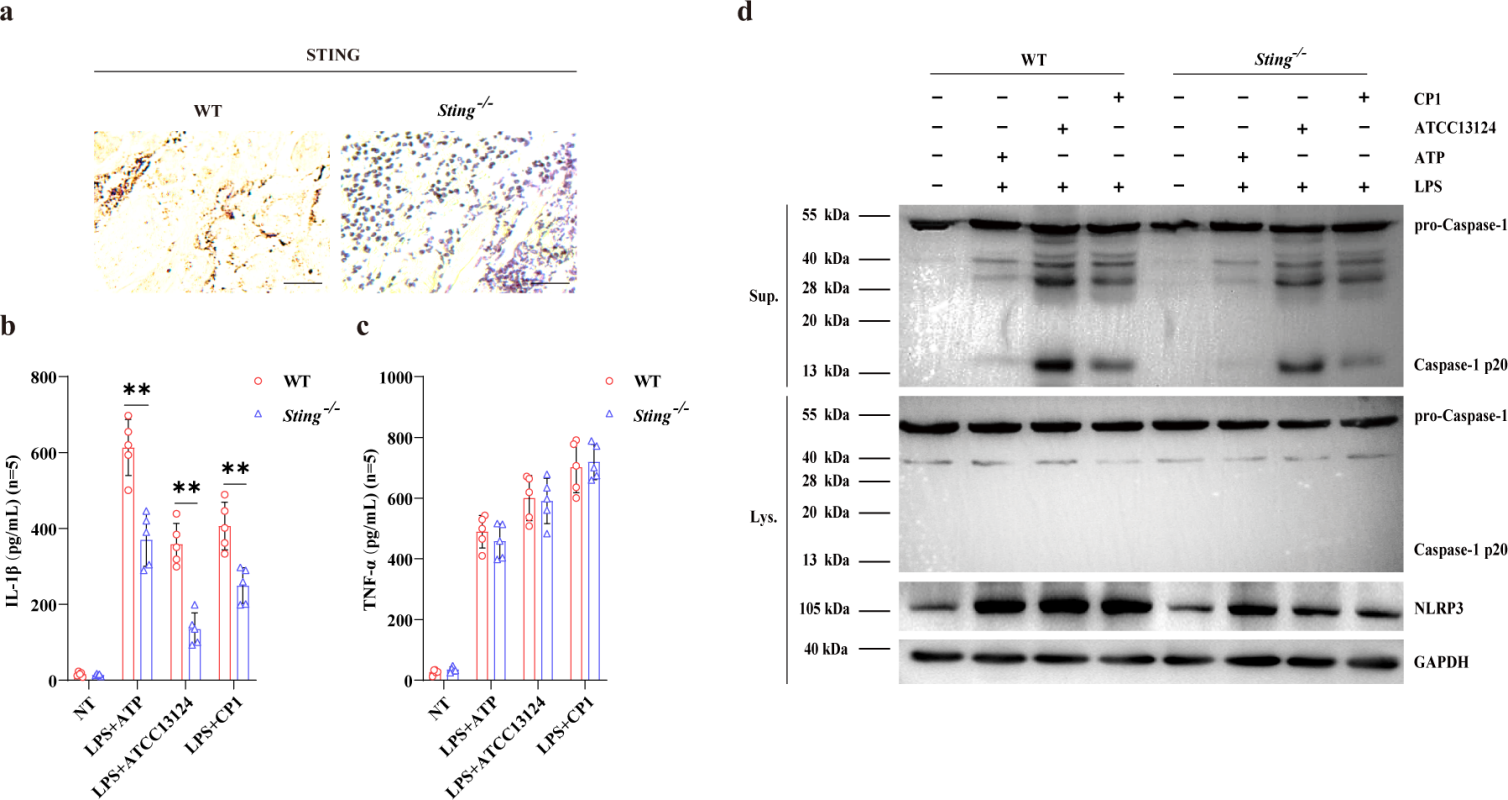
STING promotes NLRP3 inflammasome signaling activation in host macrophages. Age- and sex-matched WT and *Sting*^-/-^ mice were intramuscularly infected with 1×10^7^ CFUs log-phase *C. perfringens* at 24h p.i. **(a)** Representative infected muscle sections. STING (IHC) stained brown (magnification, ×400). LPS-primed WT and *Sting*^-/-^ BMDMs were exposed to indicated concentrations/dosages of ATP (5mM, 30min) or *C. perfringens* (MOI = 20, 90 min). **(b, c)** Secreted IL-1β and TNF-α in the culture supernatant were measured by ELISA. **(d)** Cleaved Caspase-1 in culture supernatants (Sup.) or in cell lysates (Lys.), as well as NLRP3 in cell lysates were analyzed by immunoblotting. Graphs are means ± standard deviation (SD) from data pooled five **(b, c)** biological replicates. Data were considered significant when **p < 0.01.

**Figure 4:**
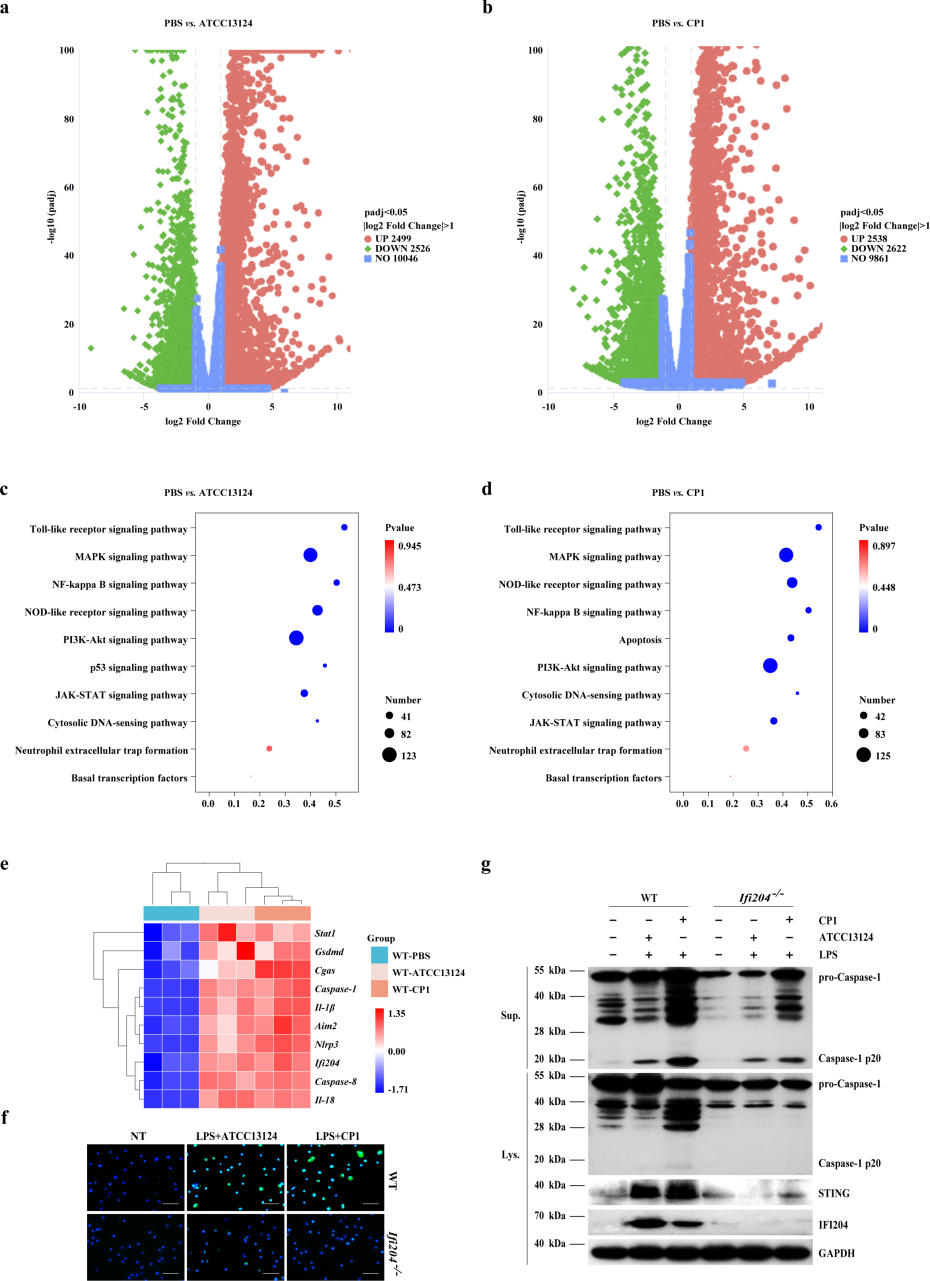
STING facilitates NLRP3 inflammasome signaling activation in an IFI204-dependent manner. LPS-primed BMDMs were exposed to indicated dosages of log-phase *C. perfringens* (MOI = 20, 90 min). Macrophages were harvested for RNA extraction and subjected to RNA sequencing. **(a, b)** Volcano plot (*C. perfringens* group vs. PBS group). **(c, d)** The top significantly upregulated KEGG pathways in *C. perfringens*-stimulated macrophages. **(e)** Top significantly regulated genes in *C. perfringens*-triggered macrophages. LPS-primed BMDMs were exposed to indicated dosages of log-phase *C. perfringens* (MOI = 20, 90 min). **(f)** IFI204 expression was assessed by immunofluorescence (magnification, ×400). **(g)** Cleaved Caspase-1 in culture supernatants (Sup.) or in cell lysates (Lys.), as well as STING and IFI204 in cell lysates were measured by immunoblotting.

Since immunohistochemical staining showed that STING expression was mainly located in the infiltrated inflammatory cells of the infected muscles during gangrenous *C. perfringens* infection (**Figure 3a**), and STING expression was strikingly up-regulated by bacteria administration in BMDMs (**Figure 4g**; **Supplementary Figure 3**), we then further substantiate the inflammatory pathways in macrophages *in vitro*. In line with *in-vivo* findings, the inflammasome signaling was significantly suppressed in infected *Sting^-/-^* BMDMs compared to infected WT BMDMs when exposed to pathogenic *C. perfringens*, observed by restricted Caspase-1 activation and impaired IL-1β release, while TNF-α production was little affected (**Figures 3b–d**; **Supplementary Figure 2**). Importantly, NLRP3 expression was markedly attenuated in *Sting^-/-^* BMDMs *versus* WT BMDMs (**Figure 3d**; **Supplementary Figure 2**). These results demonstrated that *Sting* deficiency leads to the defect of NLRP3 inflammasome signaling activation following pathogenic *C. perfringens* challenge.

### STING promotes NLRP3 inflammasome-mediated bacterial killing dependent on IFI204 signaling

To identify which defenses and networks may be associated with STING-mediated NLRP3 inflammasome signaling activation, we conducted RNA sequencing (RNA-seq) analysis to obtain genome expression differences in BMDMs infected by *C. perfringens*. Based on the threshold criterion of log_2_ Fold Change > 1 and p < 0.05 significance, the analysis screened 2,499 upregulated and 2,526 downregulated genes in *C. perfringens* strain ATCC13124-stimulated macrophage, and 2,538 upregulated and 2,622 downregulated genes in *C. perfringens* strain CP1-stimulated macrophages (**Figures 4a, b**). KEGG enrichment analysis further highlighted the upregulation of the NOD‐like receptor signaling pathway, NF-kappa B signaling pathway, and cytosolic DNA-sensing pathway following *C. perfringens* stimulation (**Figures 4c, d**). As shown by the cluster heatmap of differential genes related to innate immune responses, *C. perfringens* infection promoted or enhanced the induction of many genes compared to uninfected controls, including Ifi204, Nlrp3, Caspase-1, Gsdmd, and Cgas (**Figure 4e**). Given that IFI204 (the murine homolog of human IFI16), a STING pathway regulator, has been previously indicated to sense pathogen infection, we set out to evaluate the functional role of IFI204 in response to pathogenic *C. perfringens*. Interestingly, immunofluorescence and Western blot analysis confirmed that the expression of the IFI204 protein level was remarkably upregulated by pathogenic *C. perfringens* challenge in BMDMs (**Figures 4f, g**; **Supplementary Figure 3**). Exhilaratingly, the induction of the STING expression was significantly abrogated in infected Ifi204^-/-^ BMDMs compared to infected WT BMDMs (**Figure 4g**; **Supplementary Figure 3**), indicating that IFI204 may be involved in STING-mediated protective responses.

**Figure 5:**
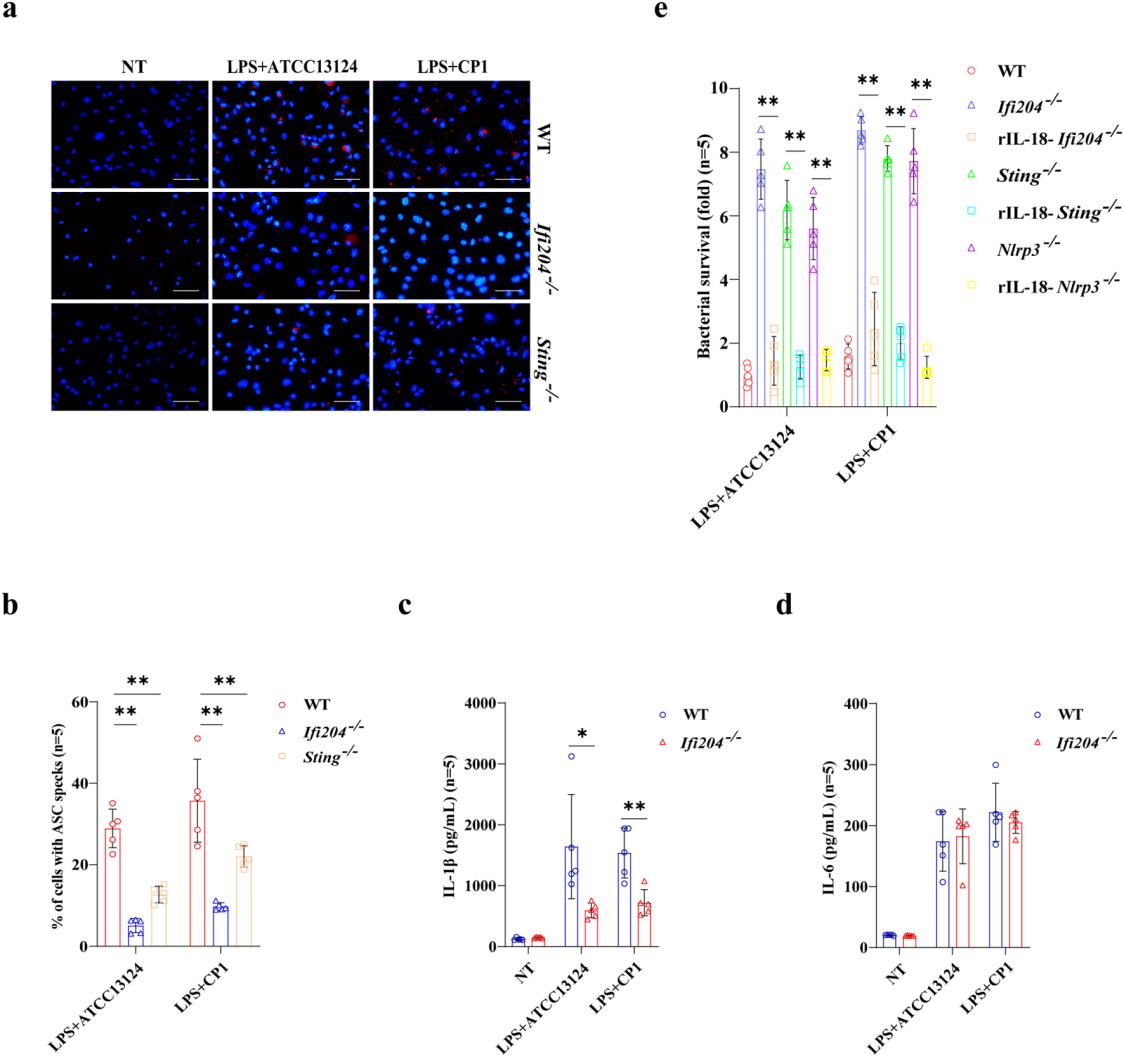
STING and *Ifi204* deficiency prevents ASC oligomerization and results in the defect of NLRP3 inflammasome-mediated bacterial killing. LPS-primed WT, *Sting*^-/-^, *Ifi204*^-/-^, and *Nlrp3*^-/-^ BMDMs were exposed to indicated dosages of log-phase *C. perfringens* (MOI = 20, 90 min). **(a, b)** ASC was determined by immunofluorescence (magnification, ×400), and the percentage of positive cells containing ASC speckles was quantified. **(c, d)** Secreted IL-1β and IL-6 in the culture supernatant were measured by ELISA. **(e)** LPS-primed WT, *Sting*^-/-^, *Ifi204*^-/-^, and *Nlrp3*^-/-^ BMDMs were incubated with rIL-18 (1,000 pg/ml) or PBS for 1h prior to were exposed to indicated dosages of log-phase *C. perfringens* (MOI = 5, 6h). The bacterial killing was assessed. Graphs are means ± standard deviation (SD) from data pooled five **(b–e)** biological replicates. Data were considered significant when *p < 0.05 or **p < 0.01.

Subsequently, we asked if IFI204 regulated STING-mediated NLRP3 inflammasome signaling. Similarly to that of infected Sting^-/-^ BMDMs, Caspase-1 cleavage, ASC speckles formation, and mature IL-1β secretion were abolished in infected Ifi204^-/-^ BMDMs, although IL-6 production was little affected (**Figures 4g**, **5a–d**; **Supplementary Figure 3**). Crucially, Sting deficiency, Ifi204 deficiency, and Nlrp3 deficiency impaired the bacterial killing capacity of BMDMs, and exogenous rIL-18 administration could rescue the defect of bacterial killing in Sting^-/-^ BMDMs, Ifi204^-/-^ BMDMs, and Nlrp3^-/-^ BMDMs (**Figure 5e**). Indeed, our previous studies have reported that NLRP3 inflammasome signaling activation is beneficial to strengthening bacterial control and host defense following pathogenic *C. perfringens* infection (12, 25). These findings suggested that IFI204 is a crucial regulator of STING signaling for activating antibacterial innate immunity.

### IFI204 is required for STING-mediated host defense

**Figure 6:**
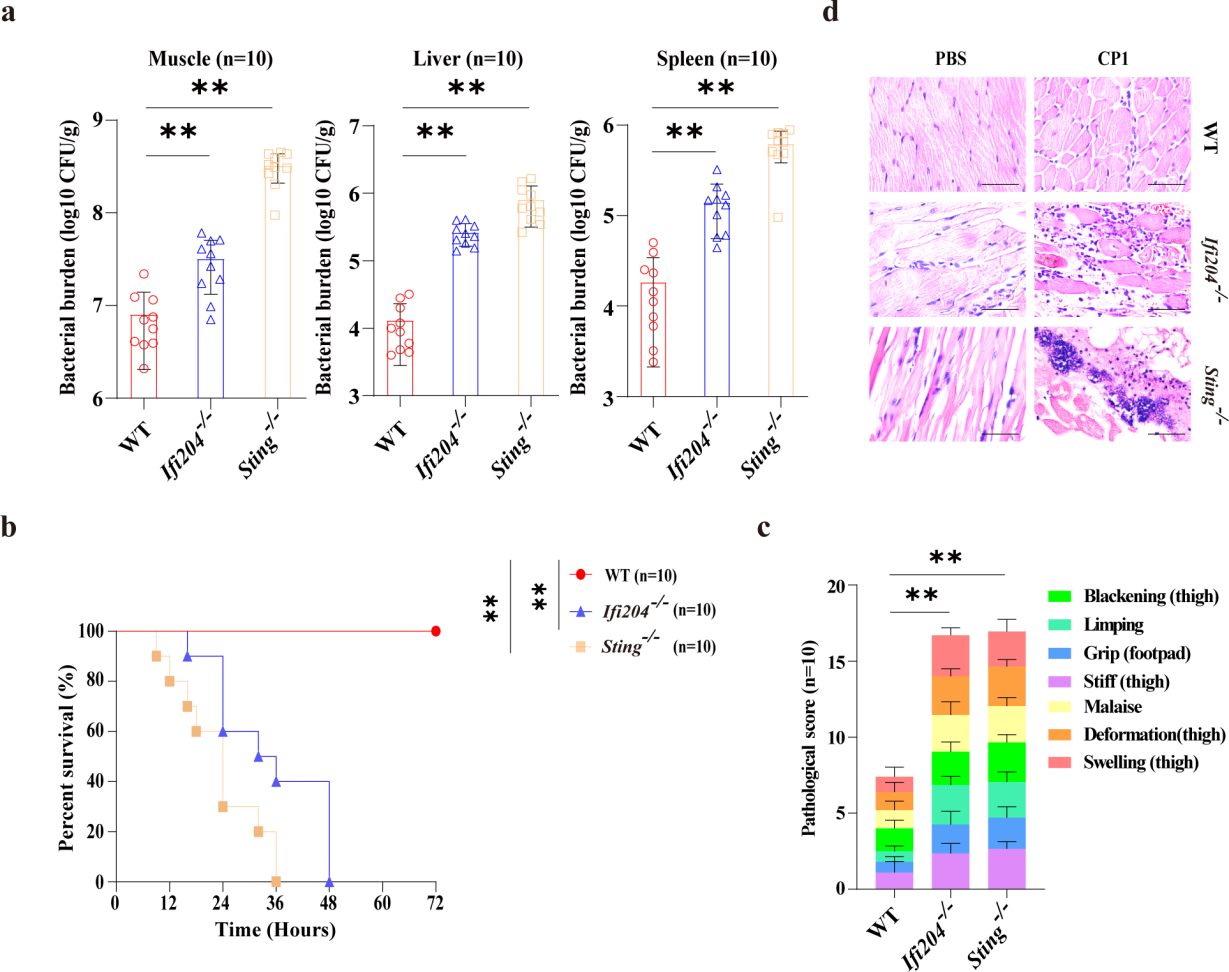
*Ifi204* deficiency is insufficient to drive bacterial clearance and host defense against gangrenous *C. perfringens* infection. Age- and sex-matched WT, *Sting*^-/-^, and *Ifi204*^-/-^ mice (*n* = 10 each group) were intramuscularly challenged with log-phase *C. perfringens*. **(a)** Bacterial loads in the muscle, liver, and spleen were enumerated (1×10^7^ CFUs, at 24h p.i.). **(b, c)** Survival and cumulative gross pathology scores of infected mice (5 × 10^7^ CFUs). **(d)** Representative H&E staining of the leg muscle tissue (1 × 10^7^ CFUs, at 24h p.i., magnification, ×400). Graphs are means ± standard deviation (SD) from data pooled ten **(a–c)** biological replicates. Data were considered significant when **p < 0.01.

To further identify the underlying signaling mechanisms that IFI204 regulates STING-NLRP3 signaling-driven host defense, *Ifi204^-/-^* mice were utilized to characterize the effect of IFI204 on pathogenic *C. perfringens* gas gangrene. Excitedly, in line with *Sting^-/-^* mice, *Ifi204^-/-^* mice are more susceptible to gangrenous *C. perfringens* infection compared with WT counterparts, demonstrated by boosted bacterial numbers observed in muscle, liver, and spleen (**Figure 6a**), indicating that *Ifi204* deficiency is also detrimental to bacterial clearance. Correspondingly, *Ifi204^-/-^* mice displayed higher mortality rates, obvious clinical symptoms, and severely impaired muscle architecture (**Figures 6b–d**), suggesting that IFI204 is essential for host protection against pathogenic *C. perfringens* soft tissue infection.

**Figure 7:**
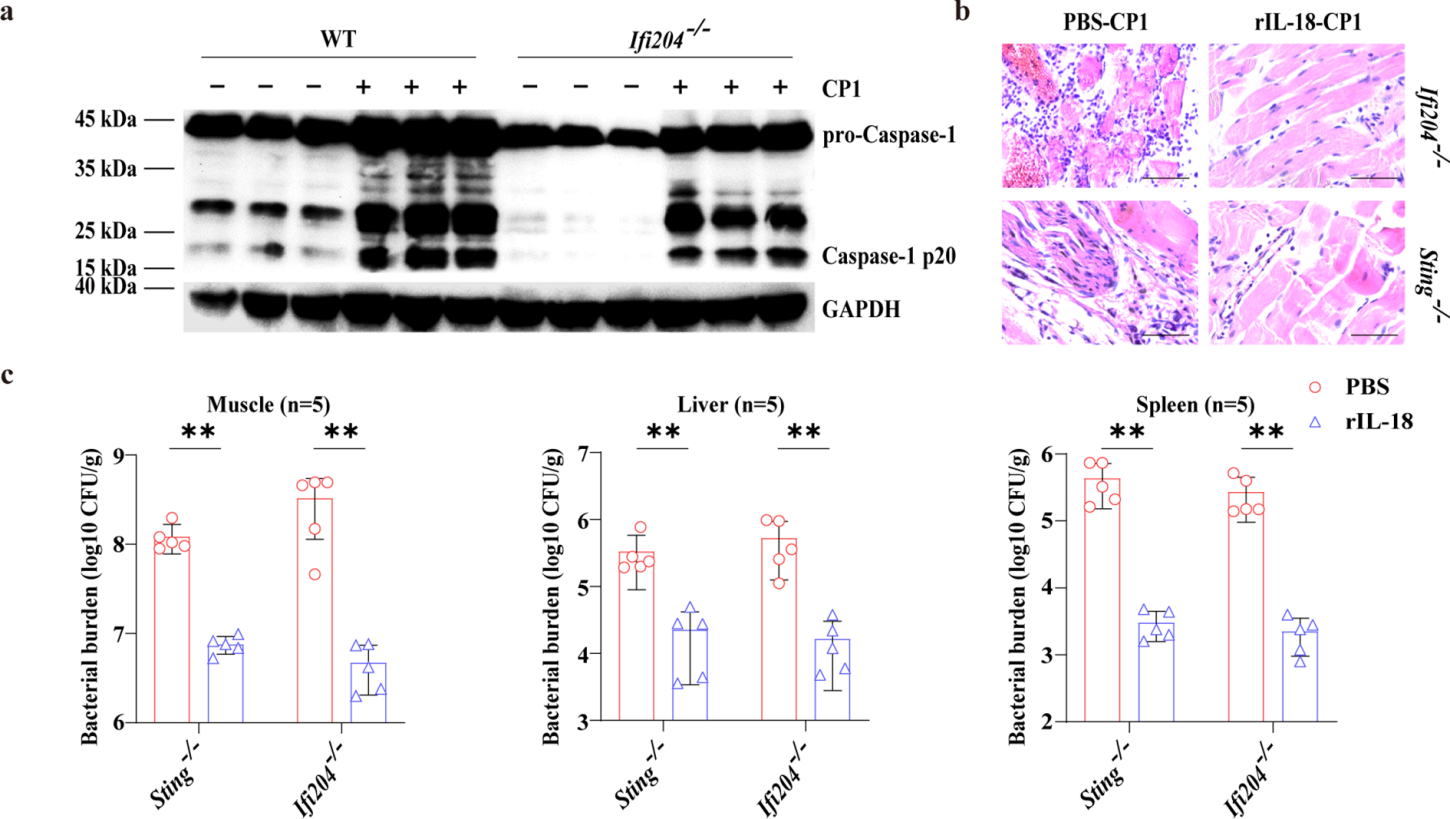
NLRP3 Inflammasome signaling downstream of IFI204-STING axis confers protection against gangrenous *C. perfringens* infection. Age- and sex-matched WT and *Ifi204*^-/-^ mice were intramuscularly challenged with 1 × 10^7^ CFUs log-phase *C. perfringens* at 24 h p.i. **(a)** Cleaved Caspase-1 in the muscle tissue lysate was examined by immunoblotting. For one group of *Ifi204*^-/-^ or *Sting*^-/-^ mice, rIL-18 (1 μg/mouse) was injected intraperitoneally daily starting the day before the intramuscularly infected with 1 × 10^7^ CFUs log-phase *C. perfringens* at 24h p.i. **(b)** Representative H&E staining of the leg muscle tissue (magnification, ×400). **(c)** Bacterial loads in the muscle, liver and spleen were enumerated. Graphs are means ± standard deviation (SD) from data pooled five **(c)** biological replicates. Data were considered significant when **p < 0.01.

Subsequently, to determine if IFI204-mediated inflammasome activation was involved in protection against *C. perfringens* infection. Obviously, *Ifi204* deficiency dramatically abrogated inflammasome-dependent Caspase-1 activation and aggravated pathological damage in the muscle (**Figures 7a, b**; **Supplementary Figure 4**). Meanwhile, infected *Ifi204^-/-^* mice and infected *Sting^-/-^* mice administered with rIL-18 exhibited strongly decreased loads of gangrenous *C. perfringens* in the muscle, liver, and spleen, and ameliorated pathological damage in the muscle (**Figures 7b, c**). Nearly the same results can be found in the results of the rIL-18–treated macrophages (**Figure 5e**), indicating that *Ifi204* and *Sting* deficiency lead to a defect in bacterial killing and clearance by impairing NLRP3 inflammasome activation. Collectively, our findings suggest that STING plays a scaffolding role in enhancing the IFI204-STING-NLRP3 signaling pathway that confers protective innate immunity against gangrenous *C. perfringens* infection.

## Discussion

*C. perfringens* is the most common cause of trauma-associated necrotizing gas gangrene with increasing mortality rates. Radical amputation and broad-spectrum antibiotic drugs are recommended choices for the treatment of this pathogenic infectious disease (44, 45). However, antibiotic use has been associated with adverse outcomes, including encouraging the spread of resistance and increasing the prevalence of drug-resistant strains. Given the crucial role of innate immunity in host protection against pathogenic infections, improving its response may be a potential prevention strategy for *C. perfringens* infections. Here, this observation highlights the importance of STING in generating host-protective innate immunity to restrict *C. perfringens* soft tissue infection.

STING is a central molecule to activate innate immunity during infection (46). The early innate immune response to Carbapenem*-*resistant *A. baumannii* infection is activated in a cGAS-STING-dependent manner (47). Moreover, STING is reported to be crucial for the host defense against *S. aureus* infection through suppressing necroptosis (36). Although the study has reported STING-dependent trained immunity contributes to host resistance to *C. perfringens* infection through the mTOR signaling pathway (48), the biological implications of STING-regulating innate immune response remain poorly understood. Using the gas gangrene model, we elucidated that compared with WT mice, *Sting^-/-^* mice were more susceptible to *C. perfringens* infection, manifested by higher bacterial loads, enhanced inflammatory cell recruitment, increased mortality, and severe damage to the muscle architecture. These results indicate that STING plays a critical protective role against *C. perfringens* soft tissue infections.

Subsequently, we investigated the immune response mechanisms of STING in defending against *C. perfringens* infection. We found increased PMN infiltration, elevated KC expression, and attenuated inflammasome signaling activation in the muscle tissue of infected *Sting^-/-^* mice relative to infected WT mice. These findings demonstrate that *Sting* deficiency disrupts inflammatory homeostasis in response to *C. perfringens*–induced gas gangrene pathogenesis. Notably, we observed that STING was predominantly expressed in inflammatory cells and significantly induced in BMDMs during *C. perfringens* infection. Therefore, macrophages were further explored as a model for *in-vitro* studies. Consistent with *in-vivo* data, we have found that *Sting* deficiency significantly impaired *C. perfringens*–induced NLRP3 inflammasome signaling, demonstrated by eliminated Caspase-1, reduced mature IL-1β release, and decreased NLRP3 expression. Indeed, inflammasome signaling is implicated in *C. perfringens* infections. Although recent evidence shows that PFO exacerbates myonecrosis through NLRP3-dependent signaling (23), our results suggest that MLKL and GPR120 promote bacterial killing and clearance, enhancing NLRP3 inflammasome signaling during *C. perfringens* infection (12, 25). Meanwhile, our previous report describes that the AIM2 inflammasome contributes to host protection against *C. perfringens* gas gangrene (12, 22). It is therefore imperative that research explore whether STING-mediated anti-infection is dependent on inflammasome signaling during pathogenic *C. perfringens* infection. Since IL-18 has been identified as a downstream molecule of the inflammasome signaling activation, and the administration of exogenous IL-18 has been reported to mitigate *Salmonella, M. tuberculosis* and *C. perfringens* colonization (12, 49, 50). Exhilaratingly, *Sting* deficiency impaired the bacterial killing capacity, and rIL-18 treatment could rescue the defect of bacterial-killing in *Sting^-/-^* BMDMs. It has been shown that STING protects against *C. perfringens* infection by enhancing inflammasome activation.

It was reported that STING-mediated type I IFNs are involved in the innate immune response (51). Obviously, evidence has shown that STING-mediated IFNs promote host survival via sensing *S. pyogenes*–derived c-di-AMP (52, 53). Moreover, STING alleviates *P. aeruginosa–*induced pulmonary inflammation via cGAS-STING-IFNs signaling (54). In addition to cGAS, IFI204 senses dsDNA during *F. novicida* infection to elicit STING activation and the type I IFN response (55). Based on these studies, to elucidate the upstream signaling of STING activation during *C. perfringens* infection, the transcriptome assay was performed. The results show that the expression of *Ifi204* was significantly upregulated in infected BMDMs. Simultaneously, we found that *Ifi204* deficiency substantially inhibited STING accumulation during *C. perfringens* infection, suggesting that IFI204 may be involved in STING-mediated host defense. Indeed, studies have shown that IFI204 promotes the STING-IRF3 pathway and enhances bacterial clearance during *S. aureus* infection (56). Obviously, similarly to *Sting^-/-^* macrophages, significant inhibited Caspase-1 activation, reduced ASC speckles formation, and attenuated bacteria-killing capacity were seen in *Ifi204^-/-^* macrophages compared with WT macrophages. Based on these findings, it implies that STING-driven NLRP3 signaling is dependent on active IFI204.

As an atypical inflammasome, IFI16 is reported to play a significant role in the innate immune response. Previous studies have shown that the IFI16 inflammasome was activated by Epstein-Barr virus, Kaposi sarcoma-associated herpesvirus, and bovine viral diarrhea virus (57–59). Furthermore, IFI16 can restrict HIV-1 replication via inhibiting the host transcription of Sp1 (60). Nevertheless, little is known about its role during bacterial infection. Subsequently, we further validated the critical defense mechanism of IFI204 in *C. perfringens* infection. At 24h p.i., similar to *Sting^-/-^* mice, *Ifi204^-/-^* mice show higher bacterial loads, increased mortality, substantial pathological damage, and dramatically impaired Caspase-1 activation compared with WT mice. Importantly, rIL-18 administration is able to rescue the susceptibility of *Ifi204^-/-^* mice. Consistently, exogenous IL-18 treatment could rescue the defect of bacterial killing in *Ifi204^-/-^*, *Sting^-/-^*, and *Nlrp3^-/-^*macrophages. Hence, it emphasizes the essential role of IFI204 in STING-mediated NLRP3 inflammasome signaling for host defense.

In summary, we identify the evidence indicating the significance of STING in an intriguing host-protective innate immune response to restrict gangrenous *C. perfringens* infection. Acting as a scaffolding hub, STING mediates the IFI204-STING-NLRP3 axis to strengthen bacterial control and host defense. Thus, these findings show that targeting STING may be an option for improving the therapy of pathogenic infectious diseases in the future.

## Data Availability

All data generated or analyzed during this study are publicly available in recognized repositories. RNA-seg data has been uploaded to the NCBI SRA (PRJNA1280697). *C. perfringens* strain CP1, a clinical isolate of *C. perfringens*, specific16S rDNA gene sequence data deposited at NCBI GenBank (MW440585).
