# Supplementary material for: IFI204-STING drives protective innate immunity against gangrenous Clostridium perfringens infection via regulation of NLRP3 signaling
Source: Front Immunol. 2026 Mar 31;17:1715595. doi: 10.3389/fimmu.2026.1715595 (PMC13076142; doi:10.3389/fimmu.2026.1715595)

Supplementary Uncropped Blot Images

**IFI204-STING drives protective innate immunity against gangrenous *Clostridium perfringens* infection via regulation of NLRP3 signaling**

Ming-Yue Zhang^1, †^, Jia-Qi Li ^1, †^, Qian Xu ^1, †^, Zi-Jian Zhuang^1^, Jing Liang^1^, Ao-Bo He^1^, Shu-Xin Zhang^1^, Yi Zhao^1^, Xue Chen^1^, Zhen-Yu Li^1^, Ping Sheng^1^, Yang Liu^2^, Shui-Xing Yu^1,^*

^1^ State Key Laboratory of Reproductive Regulation and Breeding of Grassland Livestock, College of Life Sciences, Inner Mongolia University, Hohhot 010070, China;

^2^ Institute of Animal Husbandry, Inner Mongolia Academy of Agricultural and Animal Husbandry Sciences, Hohhot, China.

* Corresponding authors: Address correspondence to Shui-Xing Yu, [shuixingyu@imu.edu.cn](mailto:shuixingyu@imu.edu.cn), State Key Laboratory of Reproductive Regulation and Breeding of Grassland Livestock, College of Life Sciences, Inner Mongolia University, Hohhot 010070, China.

1. Uncropped blot image for Figure 2e.


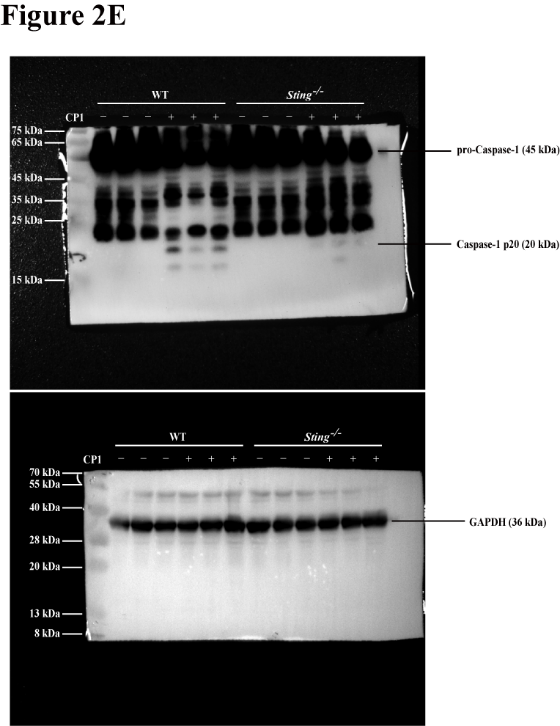


2. Uncropped blot image for Figure 3d.


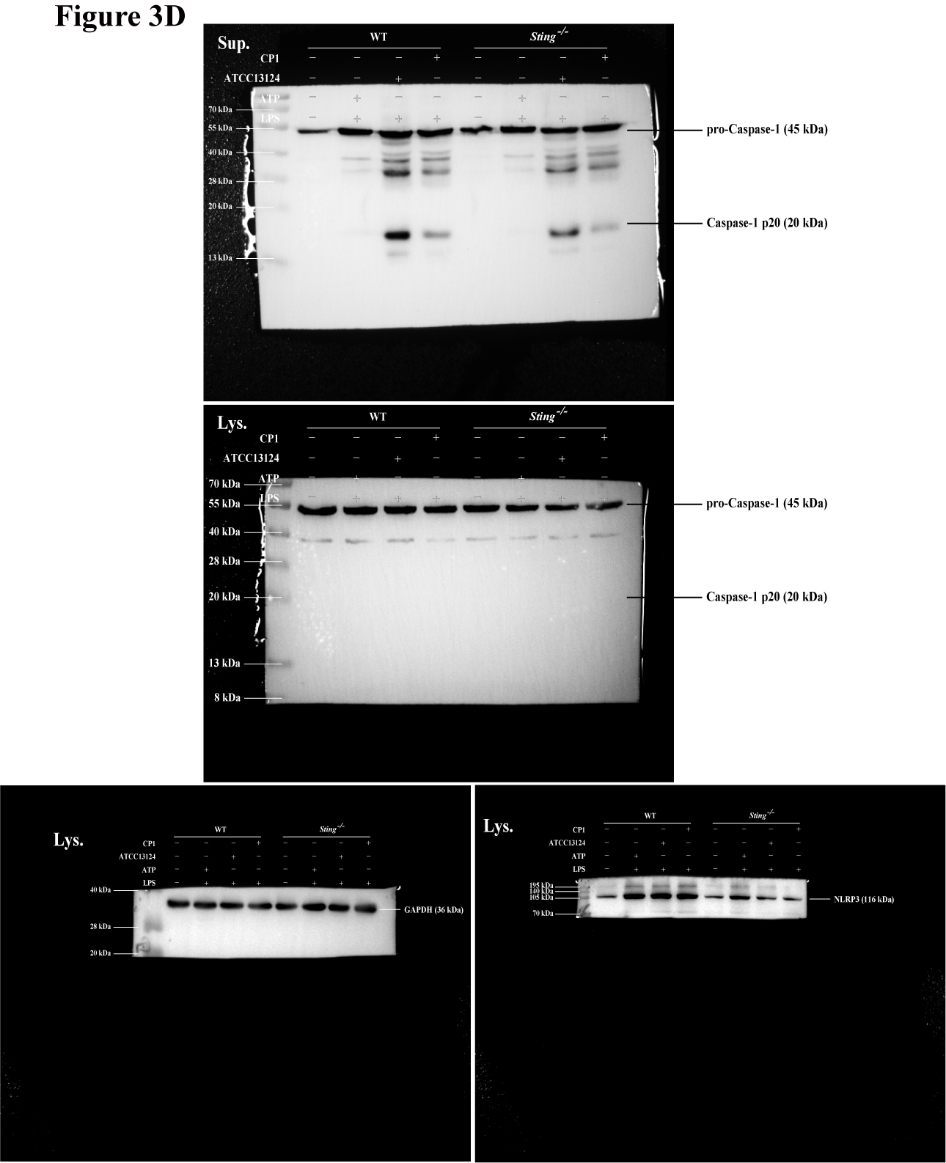


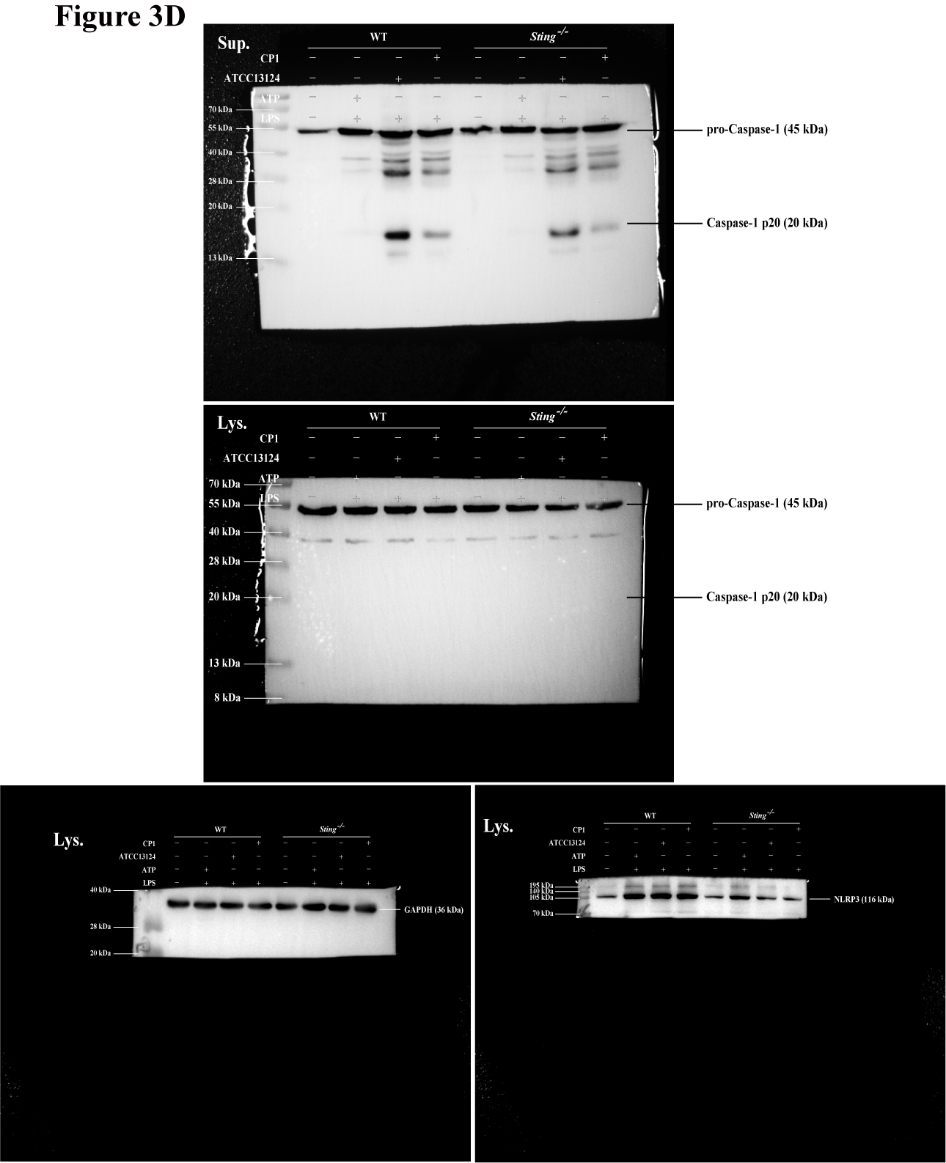


3. Uncropped blot image for Figure 4g.


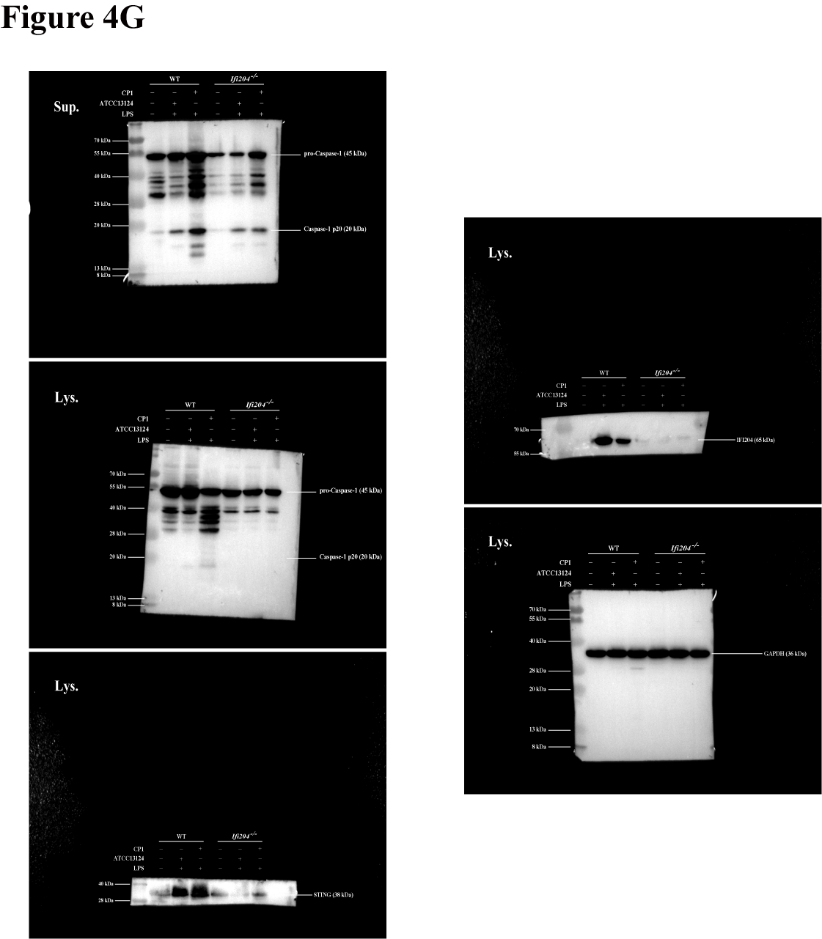


4. Uncropped blot image for Figure 7a


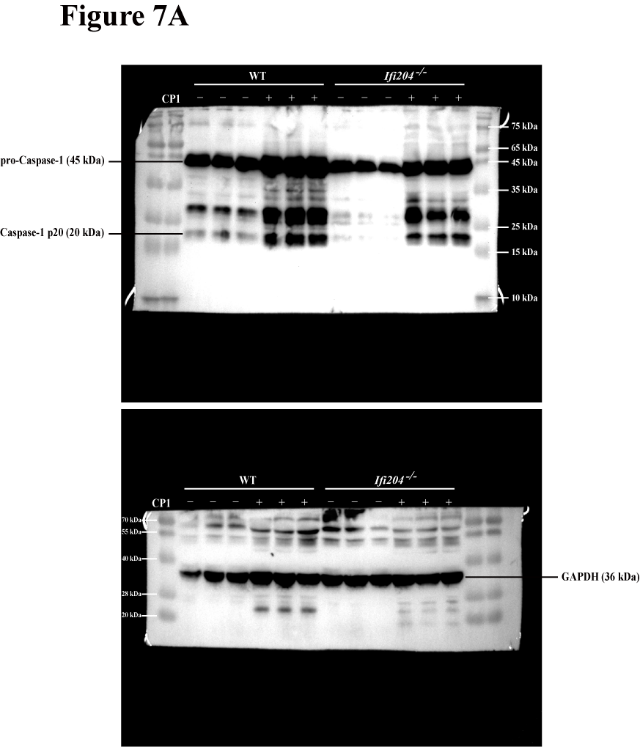

Supplement: Supplementary file 1 [file DataSheet1.docx]
