# Supplementary material for: IFI204-STING drives protective innate immunity against gangrenous Clostridium perfringens infection via regulation of NLRP3 signaling
Source: Front Immunol. 2026 Mar 31;17:1715595. doi: 10.3389/fimmu.2026.1715595 (PMC13076142; doi:10.3389/fimmu.2026.1715595)
Supplement: Supplementary file 2 [file DataSheet2.docx]

Supplementary material


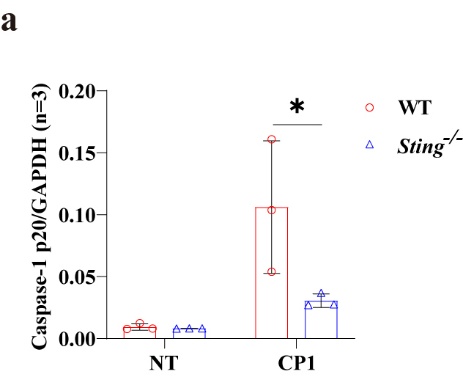


**Figure S1.** (a) amounts of Caspase-1 determined by densitometry of protein bands from three experiments.


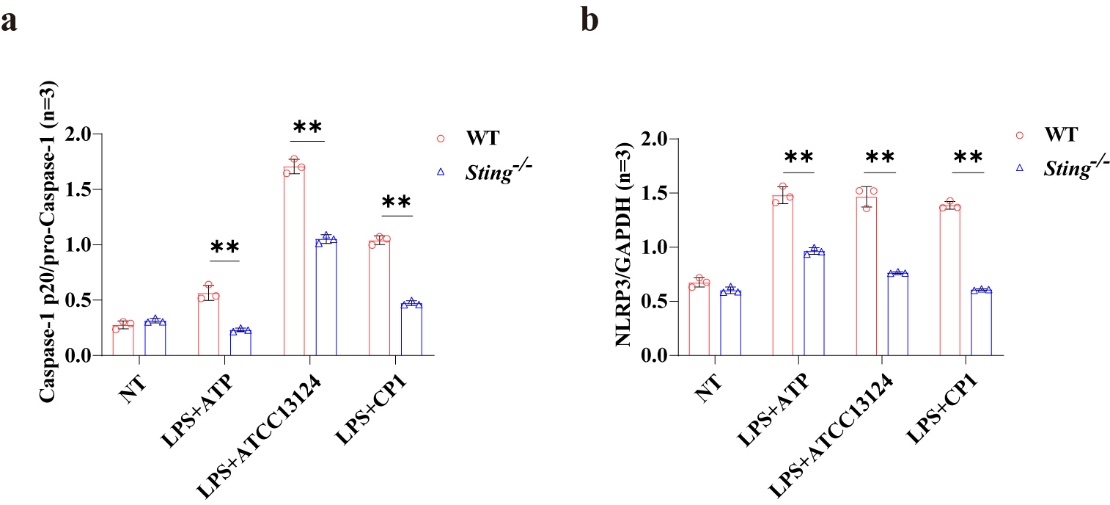


**Figure S2.** (a, b) amounts of Caspase-1 and NLRP3 determined by densitometry of protein bands from three experiments.


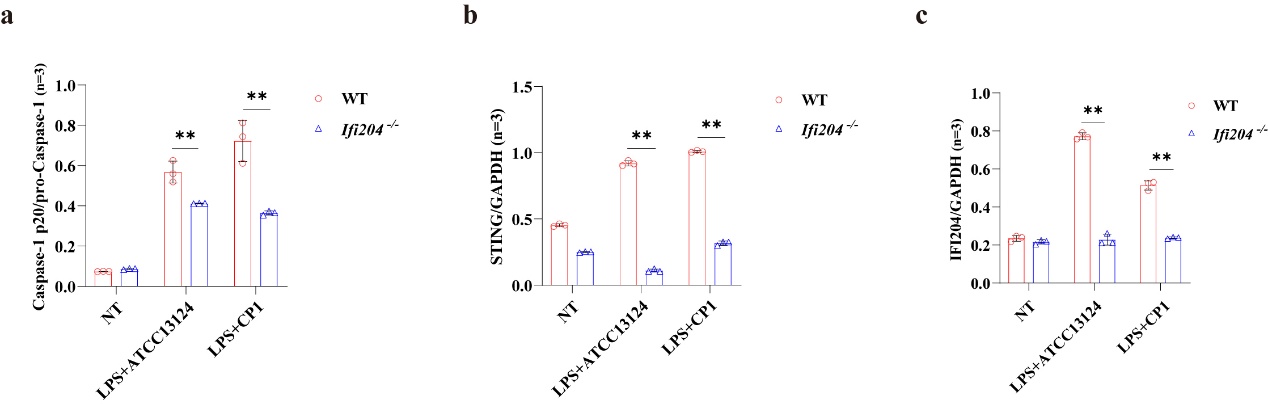


**Figure S3.** (a-c) amounts of Caspase-1, STING and IFI204 determined by densitometry of protein bands from three experiments.


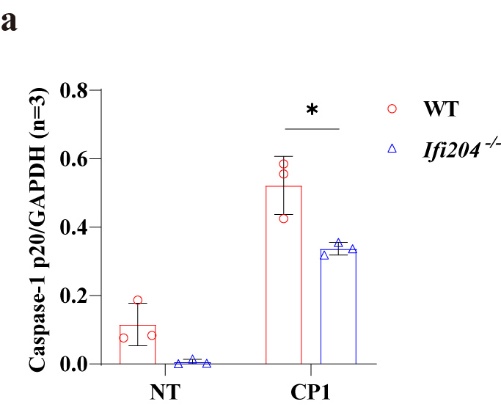


**Figure S4.** (a) amounts of Caspase-1 determined by densitometry of protein bands from three experiments.
